# EndNote 20 desktop version

**DOI:** 10.5195/jmla.2021.1260

**Published:** 2021-07-01

**Authors:** Terri Gotschall

**Affiliations:** 1 terri.gotschall@ucf.edu, Scholarly Communications Librarian, UCF Health Sciences Library, University of Central Florida College of Medicine, Orlando, FL

## Abstract

**EndNote 20 desktop version.** Released October 2020. Clarivate, 1500 Spring Garden Street, Fourth Floor, Philadelphia, PA 19130; https://endnote.com/; 1-888-418-1937; onetime purchase full license, $249.95, discounts available, contact for institutional pricing. For a list of technical requirements, visit https://endnote.com/product-details/compatibility.

Reference management software, also called citation management software, is primarily used in academia and research. All reference management software allows users to import, organize, and maintain bibliographic records [1]. There are many reference management software products on the market, for example Zotero, Mendeley, RefWorks, and EndNote. Zotero and Mendeley are free online products, and RefWorks requires a paid subscription. EndNote offers a free online version and a paid desktop version, which can be synced to the online account and allows users to share their libraries [1]. This review focuses on EndNote 20 for personal computer users, as EndNote operates differently on a Mac.

EndNote 20, an update from the version X9 desktop software, was released by Clarivate in October 2020. The update includes a major overhaul to the user interface and adds one additional function [2]. The new function is the ability to deduplicate by PubMed Central's identifier (PMCID) or the Digital Object Identifier (DOI). Deduplication of records is used heavily in systematic reviews. Bramer et al. discuss the pitfalls of using these identifiers for deduplication and instead provide a method for conducting a more effective and accurate procedure [3].

Before using the EndNote desktop version, the software must be downloaded and installed. The instructions for downloading the software will depend on whether or not the user has an individual license or if the software is provided by the user's institution. Technical specifications should be confirmed before downloading [4]. The most significant change in the technical requirements for personal computer users is that EndNote 20 requires the Windows 10 operating system. Though the software can be installed on Windows 7, users will quickly discover it does not function properly. To get the full benefit of the software, users also need to have word processing software: Microsoft Word, Apple Pages, Wolfram Mathematica 8, TextEdit, Apache OpenOffice, Nisus Writer, or Mellel [4]. Users follow the installation wizard to install the software. The last step has users create their first library or open an existing library if they are updating the software. For those updating the software, it may take a while to get used to the new workflow created by the redesign; however, importing references and using the Cite While You Write feature have remained the same.

The new user interface has removed most of the toolbar icons in favor of a less crowded toolbar area. Only five one-click icons remain: adding a new reference, sharing a group, exporting references, searching for full text, and creating a Web of Science citation report. Though most of the one-click icons have been removed, their functionality remains. For example, in version X9, a user could select a record and click on the insert reference icon to have the citation inserted into their document; with version 20, the user can still insert a reference from End-Note by selecting the record, then clicking on Tools in the menu bar, then selecting Cite While You Write, then selecting Insert Selected Citations. The added clicks are not ideal but may encourage users to use the Insert Citation feature available in the document. The option to use the keyboard shortcut Alt+2 is also available.

## IMPORTING REFERENCES

Importing references into EndNote has not changed. Users have the option to import individual or batch records from databases like PubMed and Web of Science. How to export the references varies by database, but most have some option to export references, and some offer a direct export to End-Note online or to download the RIS format. Once the RIS file is downloaded, users click on the download to open the records in the Recently Added folder in EndNote. References can also be imported from Google Scholar by clicking on the double quotation mark icon beneath the record then selecting EndNote. Again, once the file is downloaded, users click on the downloaded file to open the record in EndNote.

Users may also choose to add a new reference manually. There are over fifty reference types, including web-sites, book chapters, artwork, grants, databases, music, social media, and more. For those who have set up the free EndNote online account, there are browser extensions that scan website pages for bibliographic information to import into EndNote online, which can then be synced with the desktop version [5]. For example, in the Chrome browser there is the EndNote Capture Reference extension. Once the extension is installed, users can hover the mouse over the plug-in, and a popup will appear letting them know whether or not EndNote has access to the web-site. If it does, users click on the extension icon and a pop-up window will appear with bibliographic information autopopulated. Users will need to review the information and add anything missing before saving the record to the online account. The extension can be used to import book information from Google Books and to import all records on a page from a Google Scholar search.

## FULL TEXT OPTIONS AND ANNOTATION

Collecting and managing the full-text articles for references is a key feature in reference management software. End-Note offers several ways to attach full-text PDFs and URLs to records. Users can manually add PDFs to individual records by opening the record and clicking on the green Attach File button. Users can also perform an individual or batch record search for full text by selecting the records they want to search and clicking on the Search the Web for Full Text icon, which looks like a paper with a magnifying glass on it. Lastly, for individual records, users can install and use the EndNote Click browser extension. EndNote took over the browser extension Kopernio that allows users to find full-text articles in one click. The EndNote Click extension adds a continually spinning icon to each website page. This is not a necessary feature as it is easy to attach PDFs and URLs to EndNote records. For those with accessibility needs, the constant motion on the page can be distracting to the point where removing the extension and using one of the other two methods is preferable.

Once a PDF is attached to a record, a user can open the record, click on the PDF, and choose Open from the drop-down menu. The PDF will open in a new window, and users can add comments, highlights, search the document, print, and save the changes to the PDF.

## GROUPS

A popular feature in EndNote is the ability to create groups of references. Users can create a new group and easily click and drag records into the group. The record remains in the All References folder and can be moved from one group to another or deleted from a group without being removed from the library. Users should be aware, however, that if a record is deleted from the All References folder, it is also deleted from the group.

EndNote allows users to share groups through their online accounts with up to 100 EndNote desktop users. From the desktop version, users click on the group they want to share, then click the Share This Group icon. Users will be promoted to login or to create an account in order to sync and share. They can then choose their collaborators and assign each collaborator “read and write” or “read only” permissions.

## CITE WHILE YOU WRITE

Manually entering references in a manuscript and making sure the references are in the correct style is time consuming and tedious. If using a style that numbers the references, then editing the document and making sure the references stay in numerical order can become challenging. Thus, the best feature of EndNote is not the ability to maintain and organize resources: it is the Cite While You Write function. Cite While You Write, aptly named, means users can insert citations into text while they are writing their manuscript. End-Note inserts the selected citation in the proper style and inserts that resource into a reference list. Users can easily switch between citation styles, customize styles, and download journal-specific styles. This saves authors hours in creating and maintaining a reference list or bibliography. However, it is not 100% accurate, and all corrections must be made in EndNote in order to be reflected in the document. Users should be aware that if they make the correction solely in the document without updating EndNote, then close and reopen the document, the changes will revert back to the original EndNote content.

## FOR LIBRARIAN INSTRUCTORS

For new users, EndNote is powerful but not intuitive. Those just starting out will need instruction in order to fully use the software. For librarians who provide instruction on how to use End-Note or who maintain library guides for EndNote, the EndNote 20 tutorial videos provided by Clarivate no longer contain audio. This allows the videos to be more inclusive as it removes a language barrier for non-English speaking users. However, since the expectation of viewing a video includes hearing audio, at a minimum background music, first-time viewers may spend several minutes trying to figure out how to turn on the audio only to finally realize there is no audio. Experienced users upgrading to version 20 can follow the videos without audio instructions. This is not ideal for a new user who may need to hear the instruction while performing the task. This lack of audio instruction may make the instructional videos less usable for library guides, answering reference questions, or in instructional settings.

## ENDNOTE FOR SYSTEMATIC REVIEWS

EndNote can be a handy tool for those conducting systematic reviews. The group tools can be used to organize records from different databases. When conducting a systematic review, it is necessary to search multiple databases, often with overlapping content. For example, both Web of Science and Pub-Med search the MEDLINE database, creating duplicate records. In EndNote it only takes a few clicks to deduplicate the records, and the software provides a count of the number of records that were deduplicated, which is usually recorded in a systematic review manuscript. In addition, for users with a large number of records or who need highly accurate deduplication results, there is an additional method published by Bramer et al. that is freely accessible [3]. EndNote libraries can also be exported in several file formats that can then be uploaded to systematic review software for further analysis.

## TRAINING

EndNote is not always intuitive or easy to use, especially for new users. However, because it is widely used, a quick Google search of the issue usually results in finding an accurate solution. Many libraries have their own guides and videos that cover a wide range of issues and topics with EndNote, making it easy to troubleshoot when issues arise.

In addition, Clarivate provides extensive training on their software in multiple formats. Users have access to their full guide at https://clarivate.lib-guides.com/endnote_training/users/en20. They can also view video tutorials, guides, live sessions, and recorded webinars.

## ENDNOTE 20.0.1 UPDATE

EndNote 20 has released an update that users will want to install as it addresses a few major issues: the ability to paste by right-clicking was restored; the ability to use the delete key to move records to trash was restored; Sync log and conflict windows issues were resolved; the Find Reference Updates now updates the record; and EndNote no longer needs to be open for the direct export from a database to be used.

## CONCLUSION

Since there was limited functionality added, users who are upgrading the software will find it easy to adjust to the new workflows and decluttered interface of EndNote 20. For those who are new to using reference management software, EndNote 20 is a game changer, and users will appreciate the hours saved when they begin to use Cite While You Write.
